# Virtual Reality Simulation in Teaching Fetoscopic Laser Placental Photocoagulation in Twin‐To‐Twin Transfusion Syndrome

**DOI:** 10.1002/pd.6807

**Published:** 2025-05-01

**Authors:** Catherine Windrim, Colin Charleson, David Rojas Gualdron, Alvaro Quevedo, Lara Gotha, Tim Van Mieghem, Greg Ryan, Rory Windrim

**Affiliations:** ^1^ Ontario Fetal Centre Department of Obstetrics and Gynaecology Mount Sinai Hospital Toronto Canada; ^2^ Department of Obstetrics and Gynaecology University of Toronto Toronto Canada; ^3^ Ontario Tech University Oshawa Canada

## Abstract

**Objective:**

To develop and validate a novel virtual reality (VR) simulation system for training fetoscopic laser placental photocoagulation in twin‐to‐twin transfusion syndrome (TTTS).

**Methods:**

A VR‐based simulator incorporating Meta Quest headsets and custom‐designed hardware was developed. The system features realistic anatomical modeling, integrated performance metrics, and progressive training modules. Validation involved 31 participants (11 experienced fetal therapy specialists, 10 fetal therapy fellows, and 10 other maternal‐fetal medicine specialists) who evaluated the simulator across five domains using a standardized questionnaire.

**Results:**

The simulator demonstrated excellent internal consistency (Cronbach's *α* = 0.92) with strong positive validation across all measured aspects. Training effectiveness received the highest endorsement (87%, 95% CI: 83%–91%), followed by user engagement (85%, 95% CI: 81%–89%). Experienced specialists rated environmental realism significantly higher (4.8 ± 0.3, *p* = 0.002), while fellows provided the strongest endorsement for training effectiveness (4.8 ± 0.3, *p* = 0.004).

**Conclusions:**

This VR simulator represents a significant advancement in TTTS surgical education, offering comprehensive training capabilities without requiring practice on actual patients. Initial testing demonstrates feasibility for both local and remote teaching applications, with potential advantages in cost, portability, and educational capabilities compared to traditional physical simulators.

**Summary**

- What's already known about this topic?
  ◦ TTTS fetoscopic laser surgery requires high technical precision and extensive experience (25–60 cases) for optimal outcomes.
  ◦ Nearly half of global treatment facilities perform fewer than 20 procedures annually, creating challenges for surgical training and competency maintenance.
  ◦ Traditional latex‐based simulators, while effective, have limitations in cost, portability, and objective assessment capabilities.
- What does this study add?
  ◦ Development and validation of the first comprehensive virtual reality simulator for twin to twin transfusion syndrome fetoscopic laser surgery, integrating custom hardware with advanced virtual reality technology.
  ◦ Demonstration of strong construct validity across experience levels, with particularly high ratings for training effectiveness (87%) and environmental realism from experienced surgeons.
  ◦ Introduction of a cost‐effective portable platform enabling standardized training, objective assessment, and remote education capabilities for both individual and team‐based learning.

## 1 Introduction

Twin‐twin transfusion syndrome (TTTS) complicates approximately 15% of monochorionic twin pregnancies and, if untreated, carries high rates of mortality for both twins [1]. Fetoscopic laser ablation of placental anastamoses has been demonstrated to significantly increase survival rates for both twins and is the treatment of choice for this condition [1]. However, for educators involved in teaching the procedure there are two principal problems: firstly, it is one of the more technically challenging procedures in maternal‐fetal medicine, with significant peri‐operative fetal mortality rates. Secondly, the occurrence of TTTS is sporadic, with nearly half of all treatment facilities worldwide performing fewer than 20 procedures annually [2]. The combination of these two factors with published evidence that improved single and double survival rates for twins require that practitioners complete approximately 60 procedures and have 3.5 years of experience gives rise to a potential tension between the ability to ensure widespread access and the acquisition and maintenance of surgical competence [3, 4].

The use of simulation has been the most commonly employed tool in order to overcome these educational challenges. Our team has previously reported the development of high‐fidelity latex TTTS maternal‐fetal advanced mannequins. These simulators have been demonstrated to improve trainee performance in a randomized controlled trial and have been extensively used by fetal therapy educators over the past number of years [5, 6, 7, 8, 9, 10]. However, feedback from educators and trainees indicated that, while latex‐based TTTS simulation models offer good visual fidelity, they have practical limitations including high production costs, transportation difficulties, non‐interactivity and lack of objective performance metrics. In order to address these deficiencies, we developed a novel digital “video‐game” TTTS placental laser trainer and have demonstrated the educational promise of this serious laptop game for both in‐person and remote teaching [11, 12].

Building on our experience with this model, we identified the need for a more immersive educational platform in order to enhance the perception of operating in a real‐life surgical environment by incorporating contemporary virtual reality (VR) technology. In this report, we describe the development of a novel VR TTTS trainer and its assessment by trainees of various levels of experience.

## 2 Materials and Methods

The development of a virtual reality simulator involved significant technological advancement from our previously reported digital TTTS simulator [12]. A full‐time video‐game developer (CC) transitioned the software from the Windows Mixed Reality platform to one compatible with Meta Quest 3 headsets in order to leverage contemporary virtual reality technology [13, 14]. This migration required comprehensive redevelopment of the platform's core components, including integration of the XR (Extended Reality) Interaction Toolkit and implementation of Unity's Universal Render Pipeline [15, 16] for enhanced visual fidelity. The user interface underwent a complete redesign to optimize the virtual reality experience, incorporating VR‐native menu systems and intuitive hand tracking visualization.

**FIGURE 1:**
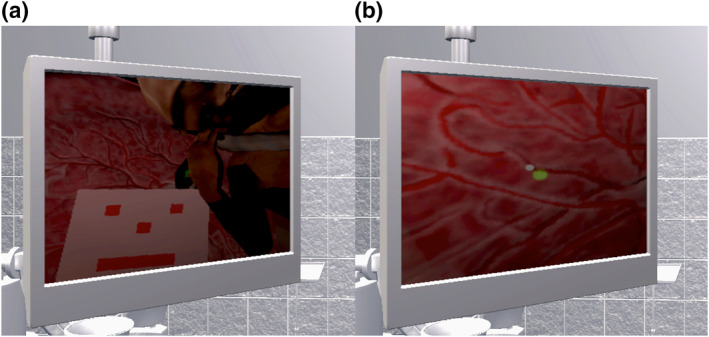
Training modes. This allows users to select if they want to train using (a) target boards or (b) the placental model. If they select training mode, the user must complete 5 different target boards that increase in difficulty as the user progresses. Each level measures the user’s accuracy and time, giving them an overall score and grade at the end of the training session. Some levels feature white circles that obscure the view of the target and should not be lasered. If the toggle is left unchecked, the user will have to complete the procedure using the placenta model.

To enhance user engagement and facilitate objective assessment, we integrated several gamification elements into the platform. These include real‐time procedure timing, live score tracking, and a comprehensive grading system. The educational framework incorporates progressive difficulty levels through multiple training modules, beginning with basic target practice (Figure 1a) and advancing to complete procedure simulation with placental models (Figure 1b). A particular focus was placed on creating hidden assessment points throughout the simulation to enable objective evaluation of procedural competency.

Additionally, the simulator employs a comprehensive assessment algorithm that utilizes procedure time and accuracy metrics to generate a live scoring system. Users progressing through the learning stages receive an overall numerical grade based on weighted performance parameters. The platform incorporates a persistent “leaderboard” function that records and displays top scores across users, introducing an element of competitive motivation within the educational framework.

**FIGURE 2:**
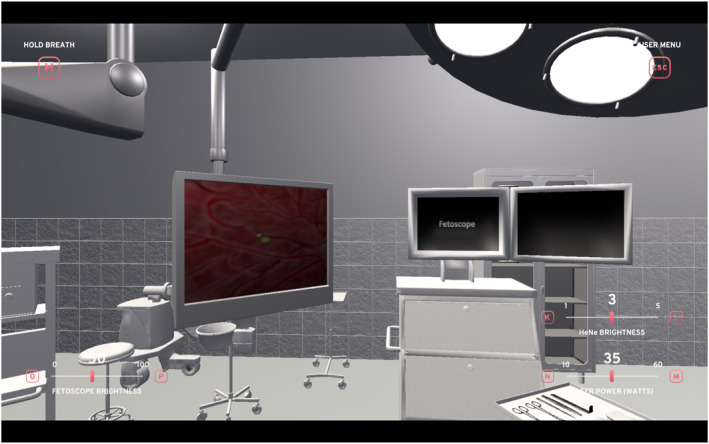
Virtual reality operating room environment. Virtual operating room setup demonstrating surgeon's perspective within the simulator. The environment includes a simulated patient with fetoscope positioning and ergonomically positioned display monitors. The interface adapts to individual user height and gaze parameters, ensuring realistic surgical orientation. The system incorporates adjustable operative controls including fetoscope illumination, HeNe aiming beam intensity, laser power (watts), and simulated maternal breathing motion with pause function to replicate maternal breath‐holding during critical steps.

**FIGURE 3:**
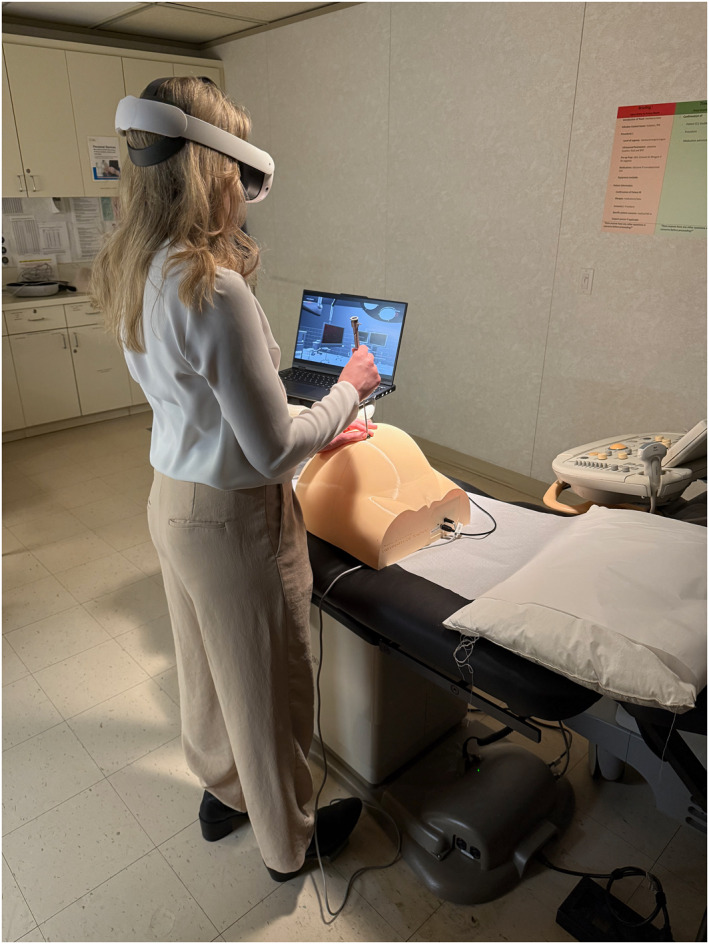
Virtual reality simulator setup. This image demonstrates a trainee using the complete VR simulator system. The trainee wears a Meta Quest 3 Headset while manipulating the custom engineered fetoscope controller. Note the 3D‐printed maternal abdomen with integrated fetoscope that provides tactile feedback for fetoscope manipulation. The foot pedal for laser activation is visible at the bottom. This integrated hardware‐software system allows for a fully immersive training experience that combines physical and virtual elements to simulate the TTTS environment.

The virtual Operating Room environment itself was created using professional 3D modeling software and implemented in the Unity development platform (Figure 2). Anatomical models were developed with input from experienced fetal surgeons to ensure accuracy. The simulation includes realistic representation of the monochorionic placenta, anastomotic vessels, and twins, with accurate tissue interaction and real‐time visual feedback during laser activation. Hardware development focused on creating a robust and portable system. The simulator consists of a custom‐designed 3D‐printed maternal abdomen with an integrated entry point, a custom‐engineered fetoscope controller incorporating multiple sensors, and a foot pedal for laser activation (Figure 3). All components were designed using polylactic acid and thermoplastic polyurethane materials housed in a protective transport case for portability.

With University of Toronto Research Ethics Board approval (UofT REB 42567), a prospective validation study was conducted using a standardized questionnaire. Participants (*n* = 31) were stratified into three groups based on their level of fetal therapy experience: (1) experienced fetal therapy specialists (*n* = 11), (2) fetal therapy fellows (*n* = 10), and (3) other maternal‐fetal medicine specialists (*n* = 10). Participants evaluated the VR simulator across five domains: training effectiveness, engagement, environment realism, skill replication and TTTS‐specific environment accuracy. Responses were collected using a five‐point Likert scale (1 = strongly disagree, 2 = somewhat disagree, 3 = neither agree nor disagree, 4 = somewhat agree, 5 = strongly agree) as shown in Table 1. Positive validation was defined as the percentage of “somewhat agree” (4) or “strongly agree” (5) responses. This questionnaire was accessed via a QR code and anonymity was maintained throughout. Internal consistency was assessed using Cronbach's alpha, and between group comparisons were performed using Kruskal‐Wallis tests.

**TABLE 1:** Standardized questionnaire used to evaluate the VR TTTS simulator across five key domains.

Domain	Questionnaire item	5‐point likert scale
Training effectiveness	The simulator improved my understanding or skill related to fetoscopic surgery.	1 = strongly disagree
User engagement	I was engaged and focused during the simulation.	2 = somewhat disagree
Environment realism	The simulated environment felt realistic.	3 = neither agree nor disagree
Skill replication	The simulator accurately replicates technical skills used in real procedures.	4 = somewhat agree
TTTS environment	The simulator accurately represents TTTS‐specific anatomy and surgical features.	5 = strongly agree

## 3 Results

The simulator setup time required less than 5 min and demonstrated robust technical performance with no malfunctions over multiple iterations. The software maintained consistent performance metrics including minimum 90 fps frame rates and low latency response (under 20 ms motion‐to‐photon). The hardware components proved durable with no breakages through repeated use.

There were 31 participants, stratified into three groups: experienced fetal therapy specialists (*n* = 11), fetal therapy fellows (*n* = 10), and other maternal‐fetal medicine specialists without formal fetal therapy training (*n* = 10). The simulator demonstrated good internal consistency across all assessment domains (Cronbach's *α* = 0.92) (Table 2). The simulator received strong positive validation (defined as “somewhat agree” or “strongly agree” responses) across all measured aspects, with training effectiveness receiving the highest endorsement (87%, 95% CI: 83%–91%), followed by user engagement (85%, 95% CI: 81%–89%) and TTTS environment reproduction (84%, 95% CI: 80%–88%).

**TABLE 2:** Simulator validation scores by domain and experience level.

Domain	Overall positive response^a^	Expert (*n* = 11)	Fellow (*n* = 10)	Other^b^ (*n* = 10)	H‐statistic	*p*‐value
Training effectiveness	87% (83–91)	4.7 ± 0.4	4.8 ± 0.3	4.2 ± 0.8	10.8	0.004
User engagement	85% (81–89)	4.6 ± 0.5	4.5 ± 0.5	4.1 ± 0.9	9.7	0.008
Environmental realism	83% (79–87)	4.8 ± 0.3	4.4 ± 0.6	4.0 ± 0.9	12.3	0.002
Skill replication	82% (78–86)	4.5 ± 0.6	4.6 ± 0.4	3.9 ± 0.9	11.2	0.004
TTTS environment	84% (80–88)	4.7 ± 0.4	4.5 ± 0.5	4.1 ± 0.8	10.5	0.005

*Note:* Values presented as mean ± SD unless otherwise noted.

^a^ Positive response defined as “somewhat agree” or “strongly agree” with 95% CI in parentheses.

^b^ Other category includes MFM specialists without formal fetal therapy training.

Kruskal‐Wallis analysis revealed significant differences between groups across all domains (*p* < 0.01). As shown in Table 2, experienced fetal therapy specialists rated environmental realism notably high (*H* = 12.3, *p* = 0.002), while fellows provided the strongest endorsement for training effectiveness (*H* = 10.8, *p* = 0.004). The MFM specialists without formal fetal therapy training provided consistently lower but still positive ratings, suggesting the simulator's potential utility for early exposure and skill development in TTTS procedures. Correlation analysis demonstrated strong relationships between environmental realism and skill replication (*r* = 0.85, *p* < 0.001), as well as between training effectiveness and user engagement (*r* = 0.79, *p* < 0.001), suggesting coherent construct validity.

## 4 Discussion

Virtual reality assisted education has demonstrated to have high levels of acceptance and effectiveness across various surgical specialties [17, 18, 19, 20, 21]. We developed this VR simulator to address specific challenges in TTTS surgical education, where technical precision and procedural expertise are required despite limited case exposure in many centers [2, 6, 10, 11, 12].

During the development process, we addressed key challenges in TTTS surgical education. Modern fetoscopic laser treatment involves several critical technical steps, including careful vessel identification and selective coagulation [22]. Trainees can practice the “sequential” laser approach where arterio‐venous connections from donor to recipient are occluded prior to other vessels. The platform also enables practice of the “Solomon” procedure technique, which has been shown to decrease the risk of postoperative twin anemia‐polycythemia sequence [23]. The implementation of standardized assessment frameworks, combined with gamification elements, provides objective measurement of skill [24, 25].

The stratification of participants into experienced fetal therapy specialists, fellows, and other maternal‐fetal medicine specialists provides insight into the simulator's utility across different stages of surgical expertise. The high ratings from experienced fetal therapy specialists, particularly regarding environmental realism (4.8 ± 0.3), validate the simulator's accuracy in replicating the surgical environment. Fellows' strong endorsement of training effectiveness (4.8 ± 0.3) supports its value in advanced procedural training. Notably, the positive but more moderate ratings from MFM specialists without formal fetal therapy training suggest the simulator's potential role in providing early exposure and fundamental skill development for those considering specialization in fetal therapy. This graduated response pattern across experience levels aligns with the simulator's intended educational framework: as an assessment tool for experienced surgeons, a focused training platform for fellows, and an introductory experience for MFM specialists exploring fetal therapy.

The VR simulator incorporates quantitative assessment capabilities through its integrated scoring algorithm, which provides immediate performance feedback based on procedural time and targeting accuracy. The system generates a comprehensive score that objectively measures technical skill parameters, allowing for performance comparison across multiple attempts and between different trainees. While this quantitative framework effectively captures execution metrics and creates engaging competition through the leaderboard functionality, the automated assessment of definitive procedural competency is not yet fully integrated. Currently, comprehensive competency determination requires educator interpretation of the performance data alongside observational assessment. Development priorities for subsequent iterations include implementing validated competency assessment algorithms derived from expert performance benchmarks, which would enable standardized determination of procedural readiness.

Cost analysis revealed significant advantages over traditional simulation methods. The complete system, including hardware, software, and transport case, can be produced for approximately 25% of the cost of traditional latex TTTS simulators. Additionally, the system requires no consumable components, reducing ongoing operational costs. A key innovation of our system lies in its integration of individual surgical training with comprehensive team‐based simulation. Through augmented reality capabilities, the entire surgical team can practice operating room setup, safety protocols, and emergency scenarios. This multidisciplinary approach addresses a critical gap in existing TTTS simulation platforms, which typically focus solely on operator skills. Furthermore, our platform's capacity for remote education opens new possibilities for collaborative surgical networks. The ability to share experiences, learn from challenging cases, and maintain skills through regular simulation practice particularly benefits surgeons in lower‐volume centers [4, 11, 26, 27].

## 5 Limitations and Future Developments

This was a pilot study of a novel simulator and has limitations. The questionnaire we employed had not been validated previously. No comparison simulator was used for comparison and while our sample size was adequate for initial validation, larger studies with more participants in each experience category would be valuable for confirming these findings. In particular, expanding the number of MFM specialists without formal fetal therapy training could provide additional insights into the simulator's utility for early‐stage learning. Current limitations of the simulation platform include the need for enhanced haptic feedback and a larger “library” of TTTS placenta simulations. While initial user feedback has been positive, larger multi‐center studies are needed to fully establish the platform's educational efficacy. Future developments will focus on developing a cloud‐based case repository where centers can share anonymized procedural recordings, challenging cases, and training scenarios. We hope that this will create a valuable resource for both training and quality improvement. Additionally, we plan to integrate artificial intelligence‐based assessment tools to provide more sophisticated feedback on surgical techniques. With minor modifications, this platform's use could also be extended for education in laparoscopic surgery.

## 6 Conclusions

This virtual reality simulator represents a significant advancement in TTTS surgical education, offering a comprehensive solution to the challenges of surgical training and competency maintenance. While further validation studies are needed, our initial experience suggests that this platform can play a crucial role in standardizing and improving TTTS surgical training. As virtual reality technology continues to advance, it provides a foundation for ongoing innovation in fetal therapy surgical education.

## Ethics Statement

This study received approval from the University of Toronto Research Ethics Board (UofT REB 42567).

## Consent

The authors have nothing to report.

## Conflicts of Interest

The Department of Obstetrics and Gynecology at the University of Toronto has applied for a patent for this simulator. T.V.M. is an Associate Editor for Prenatal Diagnosis.

## Data Availability

The data that support the findings of this study are available from the corresponding author upon reasonable request.
